# Identification of Small Molecules as Zika Virus Entry Inhibitors

**DOI:** 10.3390/ijms262110726

**Published:** 2025-11-04

**Authors:** Abhijeet Roy, Hansam Cho, Kristin V. Lyles, Wen Lu, Ming Luo, Asim K. Debnath, Lanying Du

**Affiliations:** 1 Institute for Biomedical Sciences, Georgia State University, Atlanta, GA 30303, USA; 2 Department of Chemistry, Georgia State University, Atlanta, GA 30302, USA; 3 Lindsey F. Kimball Research Institute, New York Blood Center, New York, NY 10065, USA; 4 Drug Development Program, Center for Precision Diagnostic and Therapeutic Research, Bengaluru 560100, Karnataka, India

**Keywords:** flaviviruses, zika virus, small molecule inhibitors, envelope protein, viral entry, entry inhibitors

## Abstract

Zika virus (ZIKV) caused Zika outbreaks and continues to post threats to public health. ZIKV infection may cause congenital abnormalities during pregnancy and neurological manifestations in adults. The recurrent public health threat of Zika in various geographical areas demonstrates a need for the development of effective therapeutics. Currently, there are no approved therapies for Zika. ZIKV is a single-stranded, positive-sense RNA virus, whose genome encodes three structural proteins and seven non-structural proteins. The surface envelope (E) protein is essential for host–cell recognition and viral entry; therefore, inhibition of E-mediated viral entry is a key strategy underlying antiviral treatments. Here, molecular docking-based virtual screening was used to screen small-molecule compound libraries to identify potential ZIKV entry inhibitors. Among the compounds identified, Pyrimidine-Der1 exhibited efficient inhibition of reporter ZIKV infection. The microscale thermophoresis assay confirmed its binding with the ZIKV E protein. This compound has effective inhibition of authentic ZIKV infection in a plaque inhibition assay against R103451, PAN2016, and FLR human strains (IC_50_: ~3–5 μM). Additionally, it efficiently inhibited ZIKV infection at viral entry and fusion steps of the virus life cycle in a time-of-addition assay. Overall, Pyrimidine-Der1 is a promising ZIKV entry inhibitor, warranting further optimization and evaluation.

## 1. Introduction

Zika virus (ZIKV) was initially identified in 1947 in Zika forest, Uganda [1]. It caused several large-scale outbreaks. The first major outbreak occurred on Yap Island, Micronesia in 2007, where approximately 7000 cases were reported, followed by a larger outbreak in French Polynesia in 2013, affecting an estimated 28,000 individuals [2,3]. The most severe ZIKV outbreak occurred between 2015 and 2019 in Brazil and across the Americas, during which millions of people were infected in 48 countries and territories of the Pan-American region [4].

Although ZIKV infection is often asymptomatic in adults, it can occasionally lead to severe neurological complications, including bilateral facial palsy, acute inflammatory demyelinating polyneuropathy, and Guillain–Barré syndrome [5]. Infants born to mothers infected with ZIKV during pregnancy may present with a spectrum of congenital abnormalities, including microcephaly, brain calcifications, cortical atrophy, ventriculomegaly, paroxysmal cramps, spasticity, abnormal muscle tone, and hyperreflexia [6]. Studies have reported a nearly 20-fold higher incidence of birth defects, such as congenital Zika syndrome (CZS), characterized by microcephaly and other developmental abnormalities [7]. During the Pan-American Zika epidemic, more than 4000 cases of microcephaly and related neurological disorders were documented in newborns, underscoring the devastating impact of ZIKV on maternal and child health [8]. Currently, ZIKV infection has been reported in 92 countries [9]. The World Health Organization designated ZIKV as a potential pandemic virus [10].

ZIKV is primarily transmitted to humans through the bite of infected Aedes mosquitoes, particularly Aedes aegypti [11]. However, multiple alternative routes of transmission have been documented, including sexual transmission, blood transfusion, and laboratory-acquired infections [12,13]. In addition, viral RNA has been detected in various body fluids, such as semen, urine, saliva, breast milk, and stool, suggesting the potential for diverse non-vector transmission pathways [14]. Particularly, ZIKV can be vertically transmitted from mother to fetus, leading to congenital abnormalities and developmental defects [13].

ZIKV is an enveloped virus with a positive-sense, single-stranded RNA genome of approximately 10.7 kilobases in length. The genome contains a single open reading frame that encodes a polyprotein, composed of approximately 3400 amino acids. This polyprotein is processed by both host and viral proteases, resulting in the production of three structural proteins, including capsid, precursor of membrane or membrane, and envelope protein (E), as well as seven non-structural proteins (NS1, NS2A, NS2B, NS3, NS4A, NS4B, NS5) [15].

The E protein of ZIKV is responsible for the initial attachment of the virus to the host cell membrane, a crucial step for ZIKV infection. In the mature ZIKV particle, the ectodomain of the E protein adopts a dimeric form with three domains, including domain I (EDI), domain II (EDII), and domain III (EDIII), playing diverse roles in the viral entry into host cells [16,17,18]. EDIII interacts with receptors on the host cell surface, initiating virion internalization through clathrin-mediated endocytosis. Once the virus is internalized, the E protein undergoes significant structural changes in response to the acidic environment of the endosome, exposing the fusion loop of EDII. These structural changes in the E protein drive the fusion of the viral membrane with the endosomal membrane, creating a fusion pore. Through this fusion pore, the viral RNA genome is released into the cytoplasm of the host cell, where viral gene expression and replication can occur [13,19].

There are currently no approved antiviral medications for the prevention and treatment of ZIKV [20]. One major challenge for lacking specific treatments for ZIKV infection is the strong structural similarity between ZIKV and other flaviviruses, which makes it difficult to develop drugs that are both highly effective and selective without causing cross-reactivity [21]. Since host cell entry is the first step of ZIKV infection, inhibiting entry mediated by the surface E protein is a key strategy underlying antiviral treatments, potentially achievable by small molecule inhibitors [22]. By optimizing the biological interactions with ZIKV-specific residues, small molecules can enhance target selectivity, minimize off-target effects, and improve overall antiviral specificity [23,24].

To identify ZIKV E protein-targeting entry inhibitors, we implemented a molecular docking-based virtual screening platform to screen commercially available small-molecule compound libraries. Several small-molecule compounds have been shown to inhibit ZIKV infection, among which, a compound derived from pyrimidine series (hereinafter Pyrimidine-Der1) was identified as the most promising compound that warrants further optimization and evaluation.

## 2. Results

### 2.1. Virtual Screening for Identification of Small Molecules

**Figure 1:**
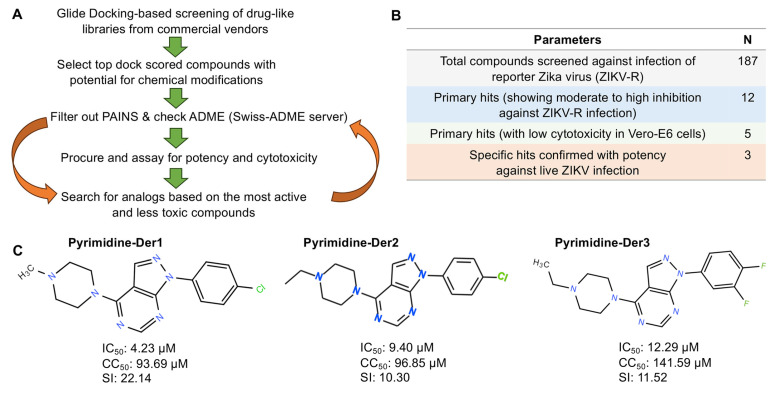
Identification of hit compounds by virtual screening. (**A**) Outline of the steps to identify hit compounds. (**B**) Small molecule compounds purchased from commercial vendors were tested against viral infection using reporter Zika virus (ZIKV-R), based on which primary hits were identified, evaluated for cytotoxicity using CCK-8 kit, and confirmed for inhibition of ZIKV infection using a plaque inhibition assay. (**C**) The identified three hit compounds were confirmed for inhibition against infection of authentic (live) ZIKV (R103451 strain) by the plaque inhibition assay. The selectivity index (SI) was calculated based on the formula of 50% cytotoxicity concentration (CC_50_)/half-maximal (50%) inhibitory concentration (IC_50_). The experiments (in (**C**)) were repeated three times, resulting in similar results.

Virtual screening was performed using Glide-based docking Schrodinger 2023 software (Schrödinger, Portland, OR, USA) to identify potential small-molecule inhibitors of ZIKV. Compound libraries from reliable commercial vendors, including Enamine, ChemDiv, and ChemBridge, were screened. Compounds with the highest docking scores were selected, filtered for PAINS (pan-assay interference structures) using the SwissADME server, and carried forward for subsequent evaluations (Figure 1A).

### 2.2. Evaluation of the Anti-ZIKV Inhibitory Activity of Selected Compounds from Virtual Screening

A total of 187 small-molecule compounds were the top hits identified from the virtual screening libraries mentioned above. Using a luciferase reporter ZIKV (hereinafter ZIKV-R)-based inhibition assay, the 187 compounds were initially screened at 10 and 50 µM against ZIKV-R infection in Vero-E6 cells. Twelve compounds exhibited moderate to strong activity (≥50% inhibition), and were identified as hit compounds (Figure 1B). These compounds were subsequently evaluated for cytotoxicity in Vero-E6 cells using the CCK-8 Cell Counting Kit. Five compounds that exhibited low or no cytotoxicity (50% cytotoxicity concentration: CC_50_ > 90 µM) were selected for further evaluation of their inhibitory activity against authentic ZIKV (R103451 strain) using a conventional plaque inhibition assay (Figure 1B). Among the tested compounds, compound Pyrimidine-Der1 exhibited the strongest inhibitory activity against ZIKV R103451 infection (50% inhibitory concentration: IC_50_ = 4.23 µM) and the highest selectivity index (SI = CC_50_/IC_50_) of 22.14 (Figure 1C). In comparison, compound Pyrimidine-Der2 and compound Pyrimidine-Der3 showed IC_50_ values of 9.40 µM and 12.29 µM, with corresponding SI values of 10.30 and 11.52, respectively (Figure 1C). These results identify Pyrimidine-Der1 as the lead compound for further evaluation.

### 2.3. Characterization of Pyrimidine-Der1 for Binding Affinity and Blockage of Antibody Binding

**Figure 2:**
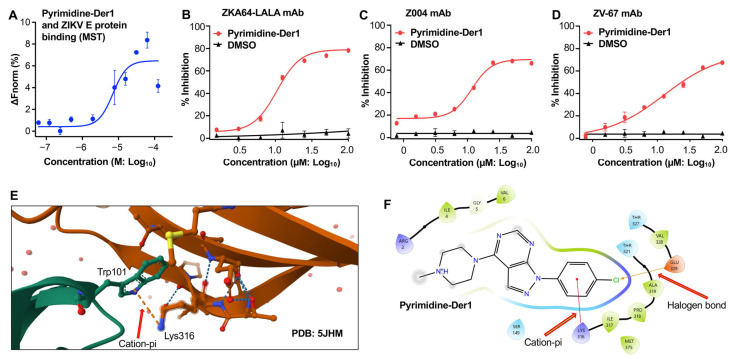
Characterization of Pyrimidine-Der1 compound for binding and inhibition ability. (**A**) The binding of Pyrimidine-Der1 to the ZIKV envelope (**E**) protein by microscale thermophoresis (MST) assay, with the calculated dissociation constant (*K_D_*) of 7.02 µM. The data is presented as the mean ± standard deviation of the mean (s.e.m) of duplicate samples. The experiments were repeated once, resulting in similar results. (**B**–**D**) Pyrimidine-Der1 inhibited the binding of ZIKV E-specific antibodies to the E protein. The plates were coated with the ZIKV E protein, and ELISA was performed to test the ability of Pyrimidine-Der1 to inhibit E-specific neutralizing monoclonal antibodies (mAbs) for binding to the E protein. The percent inhibition (% inhibition) of the compound for the binding between ZKA64-LALA (**B**), Z004 (**C**), and ZV-67 (**D**) mAbs, respectively, and the E protein was calculated in the presence or absence of the serially diluted compound based on the OD450 values, with the 50% inhibitory concentration (IC_50_) of 10.52 µM, 11.67 µM, and 12.62 µM, respectively. The data (in (**B**–**D**)) is presented as the mean ± s.e.m of duplicate wells of one experiment. The experiments (in (**B**–**D**)) were repeated three times, resulting in similar results. (**E**,**F**) Predicted binding pose (GLIDE Docking) of one of the active ZIKV entry inhibitors, Pyrimidine-Der1, on the Crystal structure of ZIKV envelope (**E**) glycoprotein in complex with Fab C10 mAb (PDB: 7a3u). A cation-pi interaction was observed between K316 and W101 in the dimer interface of ZIKV E protein (PDB: 5JHM) (**E**) [18,26]. We observed a similar cation-pi interaction between K316 of ZIKV E protein and the phenyl ring of Pyrimidine-Der1, and interestingly, we also noted a halogen bond between Cl of the molecule and E329 of ZIKV E protein (**F**).

An effective ZIKV entry inhibitor is expected to bind efficiently to the viral E protein. To assess this, the binding affinity of Pyrimidine-Der1 to a full-length ZIKV E protein was evaluated using a microscale thermophoresis (MST) assay [25]. As expected, Pyrimidine-Der1 was strongly bound to the ZIKV E protein in a dose-dependent manner, with a *K_D_* (dissociation constant) value of 7.02 µM (Figure 2A), demonstrating its specificity in targeting the ZIKV E protein.

To identify the potential binding region of the lead compound within the ZIKV E protein, an enzyme-linked immunosorbent assay (ELISA)-based competition assay was performed using three mAbs, including ZKA64-LALA, Z004, and ZV-67. These three mAbs were previously studied to specifically target the EDIII domain of the E protein, and potently neutralize ZIKV infection [27,28,29]. After coating the plates with the E protein, serially diluted Pyrimidine-Der1 or DMSO control was added to the plate in the presence of each mAb, and the relevant inhibitory rate was calculated based on the absorbance to identify the ability of Pyrimidine-Der1 to block the mAbs binding. Pyrimidine-Der1 potently inhibited the binding between the E protein and each of the three mAbs, and the inhibition was dose-dependent, with the IC_50_ values of 10.52, 11.67, and 12.62 µM, for binding of ZKA64-LALA, Z004, and ZV-67 mAbs, respectively (Figure 2B–D). In contrast, the DMSO control did not show inhibition of the binding of any of these mAbs tested (Figure 2B–D). These results indicate that Pyrimidine-Der1 potentially targets the EDIII region of the ZIKV E protein.

Computer modeling was then performed to predict the binding sites of Pyrimidine-Der1 in the EDIII region of ZIKV E protein. Despite the availability of several crystal structures of ZIKV E protein bound to different mAbs, identifying any druggable cavity on the E protein was difficult, because the mAb binding sites are mostly flat surfaces. We attempted to identify a druggable cavity using a recent crystal structure of ZIKV E protein (PDB: 7YW8, 2022) with no other bound molecules using the CavityPlus server [30]. Analysis of the amino acids surrounding the cavity indicated the presence of Lys316, which is highly conserved among vertebrate flavivirus E proteins except West Nile virus. The same Lysine residue was found to form a salt bridge with Asp50 located on the C10 mAb bound to ZIKV EDIII domain crystal structure (PDB: 7a3u) [26], indicating its importance to C10 mAb binding. This interaction also indicates that Lys316 is exposed to binding. One of the other observations was that Lys316 is located in the EDIII region next to the fusion loop region and forms a cation-pi interaction with Trp101 (PDB: 5JHM) (Figure 2E) [18,26]. Based on this crystal structure, Pyrimidine-Der1 formed a similar salt bridge with Lys316 and also has the cation-pi interaction of that Lysine with an aromatic ring (Figure 2F).

### 2.4. Identification of Pyrimidine-Der1 as an Effective ZIKV Entry Inhibitor

**Figure 3:**
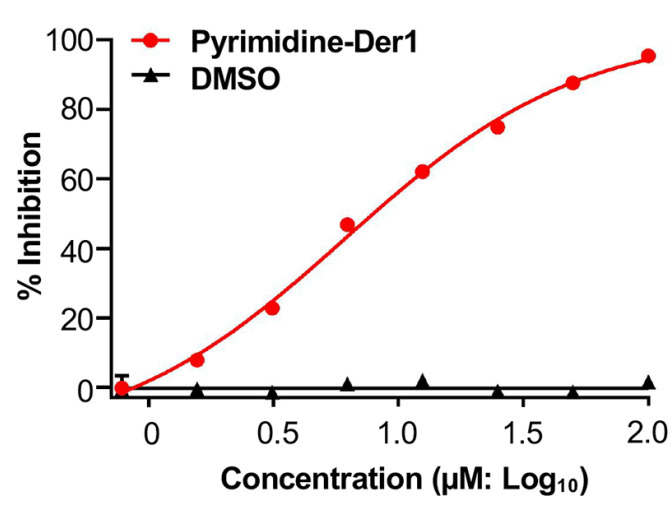
Pyrimidine-Der1 inhibited infection of ZIKV-R. The ZIKV-R was mixed with the serially diluted compound, and the mixture was added to BHK-21 cells seeded one day before experiment. After a 48 h incubation, the cells were washed and measured for relative luciferase activity. The percent inhibition (% inhibition) of Pyrimidine-Der1 against ZIKV-R infection in the cells was calculated in the presence or absence of the serially diluted compound based on relative luciferase activity, from which 50% inhibitory concentration (IC_50_: 6.13 µM) was derived. The data is presented as the mean ± s.e.m of duplicate wells of one experiment. The experiments were repeated three times, resulting in similar results.

The lead small molecule, Pyrimidine-Der1, was validated for its inhibitory activity against ZIKV-R infection in baby hamster kidney (BHK-21) cells, and related inhibitory rate was calculated from relative luciferase activity in the presence or absence of serially diluted compound, based on which the IC_50_ value was determined. Pyrimidine-Der1 inhibited ZIKV-R infection in a dose-dependent manner, with the IC_50_ value of 6.13 µM (Figure 3). In contrast, the DMSO control did not show such an inhibitory trend in the cells (Figure 3). The above data demonstrates that Pyrimidine-Der1 inhibited ZIKV-R infection in this inhibitory assay, which is reliable and convenient for rapid evaluation of its mechanism of action, as shown below.

**Figure 4:**
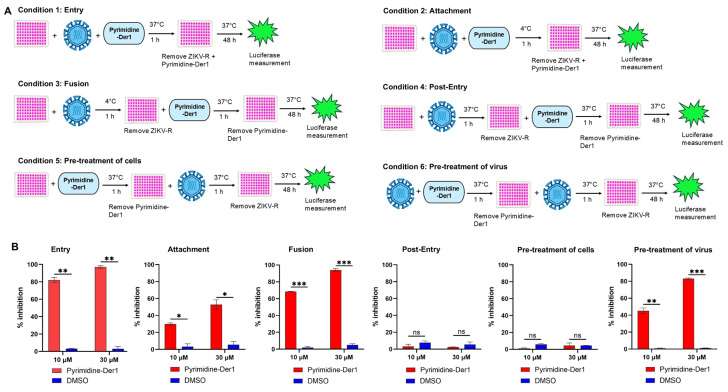
Time of addition experiment to identify potential inhibitory mechanism of Pyrimidine-Der1 to block infection of ZIKV-R. Below 6 conditions were tested (**A**). Condition 1 (Entry): Vero-E6 cells were co-treated with the virus and compound at 37 °C. Condition 2 (Attachment): The cells were co-treated with the virus at 4 °C in the presence of the compound. Condition 3 (Fusion): the cells were pretreated with the virus at 4 °C first, and then incubated with the compound at 37 °C. Condition 4 (Post-entry): The cells were pretreated with the virus, and then incubated with the compound, both of which being at 37 °C. Condition 5 (Pre-treatment of cells): The cells were pretreated with the compound at 37 °C, followed by incubation with the virus. Condition 6 (Pre-treatment of virus): The virus was pretreated with the compound at 37 °C, followed by incubation with the Vero-E6 cells. (**B**) Vero-E6 cells were infected with ZIKV-R at different conditions with or without the treatment of Pyrimidine-Der1 as described above, and the percent inhibition (% inhibition) was calculated by measuring the relative luciferase activity. The data (in (**B**)) is presented as the mean ± s.e.m of duplicate wells of one experiment. The experiments (in (**B**)) were repeated three times, resulting in similar results. Significance (*p* value) was based on the Student’s *t*-test between the Pyrimidine-Der1 and DMSO. *, **, and *** indicate *p* < 0.05, *p* < 0.01, and *p* < 0.001, respectively. ns = not significant.

We have previously established a time-of-addition assay to identify mechanisms of action of anti-ZIKV compounds against viral infection using a plaque inhibition assay [31]. Here, we adapted this assay in observing inhibitory activity of Pyrimidine-Der1 in the above-mentioned ZIKV-R inhibition assay. Specifically, six different conditions were performed (Figure 4A). By treating the compound, virus, and/or host cells under different conditions, the mechanism of action of the compound can be distinguished: blocking viral entry (Condition 1), viral attachment (Condition 2), viral fusion (Condition 3), post-entry steps (Condition 4), targeting host cells (Condition 5), and directly targeting the virus (Condition 6). (Figure 4A). Notably, Pyrimidine-Der1 (at 10 and 30 µM) had 80–100% and 70–95%, respectively, of inhibition against ZIKV-R infection at viral entry (Condition 1) and fusion (Condition 3) steps; it also showed 40–80% of inhibition of viral infection when pre-treatment of the virus with the compound (Condition 6) (Figure 4B). Additionally, Pyrimidine-Der1 inhibited 30–52% viral infection at the attachment step (Condition 2) (Figure 4B). Compared with the DMSO control, Pyrimidine-Der1 significantly inhibited ZIKV-R at the entry, attachment, and fusion steps, and blocked its infection by directly targeting the virus (Figure 4B). Instead, it did not show inhibition against other steps of viral infection, including post-entry (Condition 4), and did not directly target host cells, as demonstrated in Condition 5 by pre-treatment of cells with the compound (Figure 4B). These data shows that Pyrimidine-Der1 is a potent ZIKV entry inhibitor in blocking ZIKV entering the target cells, including the fusion step, as well as targeting the virus directly to neutralize its infection.

### 2.5. Pyrimidine-Der1 Demonstrated Broad Anti-ZIKV Inhibitory Activity

**Figure 5:**
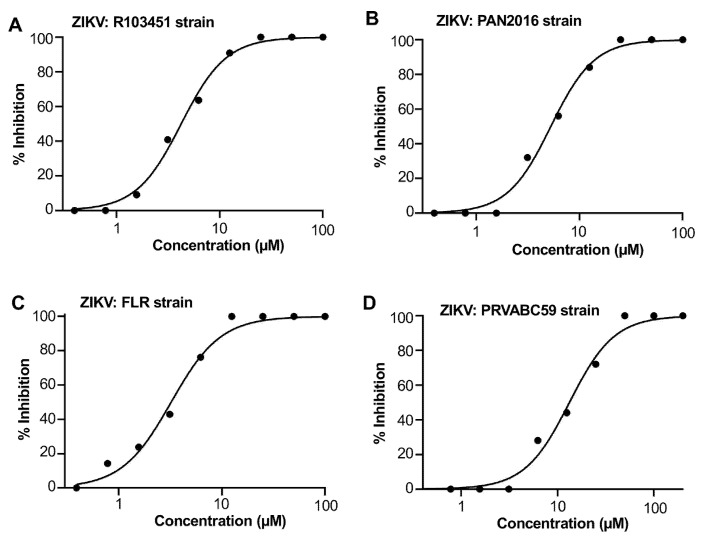
Pyrimidine-Der1 inhibited infection of different authentic ZIKV strains. A conventional plaque inhibition assay was performed to evaluate inhibitory ability of Pyrimidine-Der1 against four human ZIKV strains, including R103451 (**A**), PAN2016 (**B**), FLR (**C**), and PRVABC59 (**D**), in Vero-E6 cells, with the 50% inhibitory concentration (IC_50_) of 4.23 µM, 5.27 µM, 3.23 µM, and 13.33 µM, respectively. Each virus strain was mixed with the serially diluted compound, and the mixture was added to cells seeded one day before experiment, followed by staining plaques 5 days after infection. The percent inhibition (% inhibition) of Pyrimidine-Der1 against ZIKV infection was calculated in the presence or absence of the serially diluted compound based on the plaque numbers in 12-well plates, from which the IC_50_ was derived. The figures are representative data from one experiment. The experiments were repeated three times, resulting in similar results.

Pyrimidine-Der1 was further evaluated for its broad-spectrum ability against infection of different human strains of ZIKV, including FLR, PAN2016, and PRVABC59. The plaque inhibition assay was performed, and the relative IC_50_ values were calculated based on the inhibition rate in the presence or absence of the test compound. A strong dose-dependent inhibition of the compound was identified for all viruses tested (Figure 5A–D). In addition to inhibiting ZIKV R103451 strain, this compound potently blocked infection of FLR and PAN2016 strains (IC_50_ values ranging from 3.23 to 5.27 µM) (Figure 5A–C); it also effectively inhibited infection (IC_50_: 13.33 µM) of PAVABC59 strain (Figure 5D), a strain presenting relatively lower homology in the E protein when compared with the those of the above ZIKV strains tested. These data confirms the broad inhibitory ability of the lead small molecule against infection of different authentic ZIKV variants.

## 3. Discussion

Flaviviruses, including Dengue virus (DENV), West Nile virus, and ZIKV, continue to pose significant public health threats due to their ability to spread rapidly across large geographical regions [32]. Although promising progress has been made in the development of ZIKV vaccines, a major concern lies in the cross-reactive antibodies generated against ZIKV and other flaviviruses, particularly DENV. Evidence from in vitro studies and animal models indicates that cross-reactive antibodies may exacerbate infection through antibody-dependent enhancement, potentially worsening disease outcomes rather than providing protection [33,34]. This immunological overlap poses a significant challenge to ZIKV vaccine development and highlights the importance of alternative therapeutic strategies.

Despite global efforts to curb ZIKV transmission, treatment remains limited to supportive care [35]. The lack of targeted therapeutics underscores the ongoing need to identify and develop antiviral agents that can effectively inhibit virus transmission and mitigate disease severity. Given the rapid geographic expansion of ZIKV in recent years and its profound clinical consequences, advancing therapeutic strategies against ZIKV remains a critical priority in flavivirus research.

Small-molecule antivirals represent a valuable therapeutic approach, offering the potential to directly inhibit viral entry and replication, reduce viral load, and mitigate disease severity. Among the ZIKV treatment strategies, inhibition of ZIKV entry is a promising step for development of effective antiviral agents [22]. Therefore, the primary goal of this study was to identify small molecule compounds that inhibit ZIKV entry steps required for ZIKV to enter the host cells.

Here, we screened several commercially available small molecule libraries using viral screening based on molecular docking (GLIDE). After screening 187 top compounds, 3 specific hit compounds were identified with potency against infection of live ZIKV. Two of the hit compounds (Pyrimidine-Der2 and Pyrimidine-Der3) were discontinued due to their relatively low SI scores (10.30 and 11.52, respectively). The top hit Pyrimidine-Der1 demonstrated robust anti-ZIKV activity in the ZIKV-R inhibition assay. The compound also showed potent inhibition of ZIKV infection against different human strains of ZIKV in the plaque inhibition assay. Time of addition-based mechanistic study indicated that Pyrimidine-Der1 neutralized ZIKV by blocking the viral entry step and inhibiting the fusion step. It was also clear that Pyrimidine-Der1 directly targeted the ZIKV, rather than targeting host cells. The ELISA inhibition assay indicated that Pyrimidine-Der1 effectively bound with the ZIKV E protein, and blocked the binding of EDIII-specific mAbs. The MST binding assay revealed that Pyrimidine-Der1 bound with the ZIKV E protein in high affinity, rationalizing its anti-ZIKV activity. Computer modeling analysis suggests that Pyrimidine-Der1 interacts with an important residue of EDIII and involves the inhibition of viral entry into the host cells. Toxicity is one of the limiting factors in the therapeutic applications of many drug candidates. Pyrimidine-Der1 showed relatively low cytotoxicity with a calculated SI of 22.14. These results confirm that Pyrimidine-Der1 has the potential to be developed as an effective therapeutic agent in the battle against ZIKV.

Pyrimidine derivatives are important antiviral agents against different viruses, such as influenza, respiratory syncytial virus, rhinovirus, DENV, herpes virus, enterovirus, and hepatitis B and C [36]. These antivirals function by interfering with viral DNA or RNA synthesis, inhibiting specific enzymes, and/or interacting with key viral proteins [36,37,38]. Several other pyrimidine derivative compounds have been identified as potential entry inhibitors against ZIKV and DENV [39], further supporting the anti-ZIKV activity of Pyrimidine-Der1 reported in this study. In summary, we have identified a new class of pyrimidine derivatives as an effective entry inhibitor of ZIKV.

## 4. Materials and Methods

### 4.1. Cells and Viruses

Vero-E6 and BHK-21 cells were cultured in Eagle’s minimum essential medium (MEM) (Thermo Fisher Scientific, Waltham, MA, USA) containing 10% fetal bovine serum (FBS) (R&D Systems, Minneapolis, MN, USA) and 1% penicillin-streptomycin (Thermo Fisher Scientific). Cells were maintained at 37 °C in a humidified atmosphere of 5% CO_2_, and observed for sterility and morphology twice a week for 4–6 weeks. Human-isolated ZIKV strains, including PAN2016 (2016/Panama), PRVABC59 (2015/Puerto Rico), R103451 (2015/Colombia), and FLR (2015/Colombia), were used. Each ZIKV was cultured and maintained in Vero-E6 cells, and the plaque inhibition assay was used to determine the virus titer.

### 4.2. Virtual Screening Using GLIDE-Based Docking

We used the automated docking software GLIDE in Maestro 13.7 within Schrödinger Suit 2023 (Schrödinger, Portland, OR, USA) that applies a two-stage scoring process to sort out the best conformations and orientations of the ligand (defined as pose) based on its interactions with the receptor. GLIDE has been applied successfully in drug design [40,41,42,43,44,45]. We used the X-ray crystal structure of ZIKV E protein (PDB: 7a3u) for docking simulations to screen chemical databases from Enamine (screening libraries), ChemDiv (screening compounds), and ChemBridge Corp (Hit2Lead database). Three-dimensional coordinates of the ligands, their isomeric, ionization, and tautomeric states were generated using the LigPrep (including Ionizer) module within the Schrödinger Suite, minimized with MacroModel within Schrödinger Suite 2023 software. A grid file encompassing the cavity area containing information on the properties of the associated receptor was created. We selected the centroid of the Lys316 residue, and identified it as critical using CavityPlus 2022, a Web server, to create the grid file. We used the default grid values set in the GLIDE module within the Schrödinger Suite 2023. The conformational flexibility of the ligands was handled via an exhaustive conformational search. Initially, we used Schrödinger’s proprietary GlideScore scoring function in extra precision (XP) mode to score the optimized poses. Fifty top-scored ligands were selected from this simulation for further study.

### 4.3. Reporter ZIKV Inhibition Assay

The inhibitory ability of Pyrimidine-Der1 compound was tested with luciferase-expressing ZIKV-R [46]. The inhibitory assay was performed in BHK-21 cells. Specifically, cells (10,000/well) were seeded on 96-well flat-bottom plates one day before infection. On the day of infection, the compound at 2-fold serial dilutions were mixed with ZIKV-R with a multiplicity of infection (MOI) of 1 and incubated at 37 °C for 1 h. After incubation, the compound–virus mixture was added to the cells and cultured for 48 h. Following incubation, the cells were washed with sterile 1× PBS, and Nano-Glo substrate (diluted 1:50 in Nano-Glo Assay Buffer) (Nano-Glo^®^ Luciferase Assay System, Promega, Madison, WI, USA) was added at 50 µL per well. Luminescence was then measured using a Cytation 7 microplate reader (BioTek Instruments, Winooski, VT, USA). Percent inhibition (% inhibition) was calculated relative to the virus-only (no compound) control wells. DMSO was included as the vehicle control. Dose–response curves were generated, and the IC_50_ values were determined by nonlinear regression analysis using GraphPad Prism 10 software.

### 4.4. Plaque Inhibition Assay

Antiviral activity of Pyrimidine-Der1 compound was assessed by the plaque inhibition assay against different strains of ZIKV. Briefly, Vero-E6 cells (1.2 × 10^5^ cells/well) were seeded in 12-well plates and cultured at 37 °C 24 h before infection. On the day of infection, R103451, PAN2016, PRVABC59, and FLR strains with 25 plaque-forming unit (PFU)/well were respectively incubated with 2-fold serial dilutions of the compound or DMSO control at 37 °C for 1 h. The compound-virus mixture was then added to the cells. After incubation at 37 °C for 1 h, MEM (2 mL) supplemented with 10% FBS and 1.25% carboxymethyl cellulose (CMC) was overlaid onto the cells. After 5 days of incubation at 37 °C, cells were washed with 1× PBS, and fixed with 10% formaldehyde for 2 h at 37 °C. Finally, the cells were stained with 0.5% Crystal violet. Plaques in each well were calculated. The IC_50_ value was calculated using nonlinear regression analysis in GraphPad Prism 10 software.

### 4.5. Determination of In Vitro Cytotoxicity

The cytotoxicity of the candidate compounds was determined by the Cell Counting Kit (CCK-8) (Sigma, St. Louis, MO, USA) according to the manufacturer’s protocol. Briefly, 20,000/well of Vero-E6 cells were seeded in 96-well plates and incubated at 37 °C for 24 h. The compounds at 2-fold serial dilutions were added to the cells, and the cells were cultured for 48 h. The media was then removed, 10% CCK-8 reagent in fresh media was added to cells, and incubated at 37 °C for 1 h, followed by measurement of absorbance at 450 nm (A450 value) using a microplate reader (Cytation 7). The CC_50_ values were calculated using GraphPad Prism 10 software.

### 4.6. ELISA

ELISA was performed to determine the ability of Pyrimidine-Der1 compound to inhibit the binding of ZIKV full-length E protein-specific mAbs, including ZKA64-LALA, ZV-67, and Z004 (Absolute Antibody, Newark, CA, USA). Briefly, ELISA plates were coated with the ZIKV E protein (OPMA04848; Aviva Systems Biology, San Diego, CA, USA) at a concentration of 1 µg/mL in coating buffer overnight at 4 °C. After washing for 3 times with PBST (i.e., 1% Tween-20 in PBS buffer), the plates were blocked with blocking buffer (5% non-fat milk in PBST) at 37 °C for 2 h. The compound or DMSO (negative control) at 2-fold serial dilutions was added to the plates in the presence of each mAb at a final concentration of 0.25 µg/mL and incubated at 37 °C for 2 h. After incubation, the plates were washed 3 times with PBST, and incubated with horseradish peroxidase (HRP)-conjugated anti-human IgG-Fab antibody (1:3000) at 37 °C for 45 min. After washing 5 times with PBST, TMB (i.e., 3,3′, 5,5′-Tetramethylbenzidine) substrate was added to the plates, and the reaction was stopped by addition of H_2_SO_4_ (1 M). Absorbance at 450 nm (A450 value) was measured by the Cytation 7 microplate reader. Percent of inhibition (% of inhibition) of the mAbs by the compounds was calculated using the formula {1 − (ZIKV-E-mAb-compound/ZIKV-E-mAb) × 100}. The IC_50_ values were calculated using nonlinear regression analysis in GraphPad Prism 10 software.

### 4.7. Time of Addition Experiment

A time of addition experiment was performed to test the ability of Pyrimidine-Der1 compound to block ZIKV infection at different steps of the viral life cycle to identify the potential inhibitory mechanism [47]. This experiment was performed in Vero-E6 cells using ZIKV-R. Briefly, cells (15,000/well) and ZIKV-R in 96-well plates were incubated in the presence or absence of the compound at specific concentrations (10 µM and 30 µM) for 1 h before ZIKV-R infection, 1 h after infection, or at the same time of infection. Six conditions (i.e., Conditions 1–6) were tested. Condition 1 (Entry): cells were infected with ZIKV-R for 1 h at 37 °C in the presence of the compound or DMSO (negative control). After 1 h, cells were washed with fresh media (MEM with 10% FBS and 1% P/S) to remove unbound virus, compounds, or DMSO, and continued for culture for 48 h. Later, luciferase luminescence was measured to determine the infectivity using the above-mentioned method. Condition 2 (Attachment): cells were incubated with ZIKV-R for 1 h at 4 °C in the presence of the compound or DMSO. After washing to remove the unbound virus, compound or DMSO, the cells were cultured for 48 h, and luciferase luminescence was measured. Condition 3 (Fusion): cells were incubated with ZIKV-R for 1 h at 4 °C for viral attachment. After removal of the unbound virus, the cells were incubated with the compound or DMSO for 1 h at 37 °C for viral membrane fusion. Later, the unbound compound or DMSO was removed, and the cells were cultured for 48 h, followed by measurement of luciferase luminescence. Condition 4 (Post-Entry): cells were incubated with ZIKV-R for 1 h at 37 °C to allow the virus to enter the cells. After removing the unbound virus, the cells were incubated with the compound or DMSO for 1 h at 37 °C. The unbound compound or DMSO was removed, and the cells were cultured for 48 h, and then measured for luciferase luminescence. Condition 5 (Pretreatment of cells): cells were incubated with the compound or DMSO for 1 h at 37 °C, and the unbound compound or DMSO was removed, followed by incubation with ZIKV-R for 1 h. Later, the unbound ZIKV-R was removed, and the cells were cultured for 48 h, followed by measurement of luciferase luminescence. Condition 6 (Pretreatment of virus): ZIKV-R was incubated with the compound or DMSO for 1 h at 37 °C. 20% PEG-6000 was added, and incubated at 4 °C for 1–2 h, followed by removal of the unbound compound by centrifugation at 13,000 rpm for 1 h. ZIKV-R was then added to the cells, and the cells were incubated for 1 h at 37 °C. After removing the unbound virus, the cells were cultured for 48 h, and luciferase luminescence was further measured.

### 4.8. MST Measurement of Binding Affinity

MST measurement was carried out to estimate the relative binding affinity of Pyrimidine-Der1 compound with the ZIKV E protein. Specifically, ZIKV full-length E protein was labeled with RED-tris-NTA 2nd Generation dye (MO-L018; NanoTemper Technologies GmbH, Munich, Germany). Next, 50 nM of the protein was incubated at room temperature for 15 min, with a range of ligand (compound) concentration between 500 µM–15.3 nM. The samples were loaded onto capillaries, and the fluorescence intensity and binding affinity were measured using a Monolith X MM-333 (NanoTemper Technologies GmbH, Munich, Germany).

### 4.9. Statistical Analysis

Most experiments were carried out duplicate, and repeated one to three times to observe similar results. The IC_50_ and CC_50_ values were calculated by nonlinear regression analysis in GraphPad Software Prism 10 (Boston, MA, USA). The dose–response curve was obtained by plotting the percent inhibition against the concentration. The data is presented as the mean ± s.e.m of duplicate wells, unless otherwise indicated in the Figure legends. Student’s *t*-test was used to compare the significant difference between the Pyrimidine-Der1 and DMSO. *, **, and *** indicate *p* < 0.05, *p* < 0.01, and *p* < 0.001, respectively.

## Data Availability

Data is contained within the article. Further inquiries can be directed to the corresponding authors.

## Abbreviations

The following abbreviations are used in this manuscript:

CC50	50% cytotoxicity concentration
CZS	Congenital Zika syndrome
E	Envelope
EDI-EDIII	Envelope domains I-III
ELISA	Enzyme-linked immunosorbent assay
FBS	Fetal bovine serum
HRP	Horseradish peroxidase
IC50	50% inhibitory concentration
KD	Dissociation constant
mAb	Monoclonal antibody
MEM	Eagle’s minimum essential medium
MST	Microscale thermophoresis
NS proteins	Non-structural proteins
PFU	Plaque-forming unit
SI	Selectivity index
ZIKV	Zika virus
ZIKV-R	Reporter ZIKV
